# Pure Ocular Weakness as the Initial Manifestation of Lambert–Eaton Myasthenic Syndrome

**DOI:** 10.7759/cureus.2007

**Published:** 2017-12-31

**Authors:** Nakul Katyal, Raghav Govindarajan

**Affiliations:** 1 Neurology, University of Missouri Columbia

**Keywords:** lambert - eaton myasthenic syndrome, ocular weakness, seronegative myasthenia

## Abstract

Pure ocular presentation of Lambert–Eaton syndrome is not a common phenomenon. Such presentation poses significant diagnostic challenges and requires conscientious evaluation. In this review, we have described a case of a patient with pure ocular weakness, initially diagnosed as seronegative ocular myasthenia which on further evaluation was found to have ocular Lambert–Eaton myasthenic syndrome (LEMS).

## Introduction

Myasthenia gravis and Lambert–Eaton syndrome are autoimmune disorders characterized by defective neuromuscular transmission. Although the underlying mechanism in both conditions is related to autoantibody-mediated disruption of neuromuscular transmission, they vary significantly on clinical presentation. Ocular symptoms occur predominantly in myasthenia gravis (59% of the patients) [1], whereas Lambert–Eaton is characterized by motor weakness in proximal limbs. Pure ocular weakness as the initial clinical presentation of Lambert–Eaton myasthenic syndrome (LEMS) is uncommon. Patients with LEMS usually present with hypo/areflexia. In many patients, LEMS can coexist with other paraneoplastic syndromes. In this report, we have described a case of 59-year-old female who presented with ocular symptoms of bilateral ptosis and double vision and was initially diagnosed as having ocular myasthenia. On further workup, she was diagnosed having LEMS with underlying small cell carcinoma of lung.

## Case presentation

**Figure 1:**
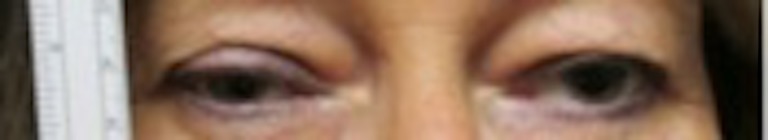
Before ice test.

A 59-year-old female presented to the ophthalmology clinic with four months history of daily headache, double vision, and droopy eyes. Her ophthalmological examination revealed bilateral ptosis (Right: 3 mm, Left: 2 mm), more pronounced in the right eye, dysconjugate gaze and double vision most appreciable on lateral gaze bilaterally (Figure 1).

**Figure 2:**
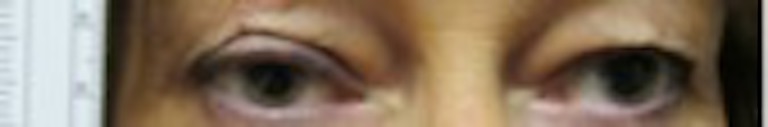
After ice test.

Ice pack test was performed on the right eye for three minutes, which showed significant improvement in ptosis (>2 mm improvement) (Figure 2).

**Figure 3:**
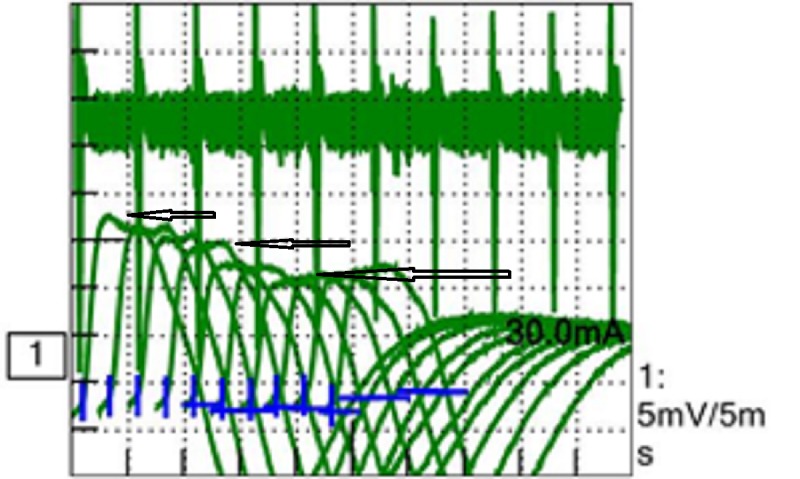
Sequential decrement in compound motor action potential (CMAP).

Acetylcholine binding/blocking and modulating receptor antibodies were negative on investigation. A working diagnosis of seronegative ocular myasthenia was established and she was referred to neurology for further evaluation. On neurological examination, her motor and sensory exam was normal. Deep tendon reflexes (DTR) were 2+ and symmetrical bilaterally. Electromyography (EMG) and nerve conduction studies (NCS) were ordered for further evaluation. On NCS of her left ulnar motor nerve, a reduced compound muscle action potential (CMAP) of 4.5 mV (Normal > 5) was noted. There was no slowing across the elbow on ulnar motor studies and ulnar sensory nerve conduction was normal. Slow repetitive nerve stimulation (at 2 Hz) of left ulnar nerve showed more than 20% decrement in CMAP amplitude (Figure 3).

**Figure 4:**
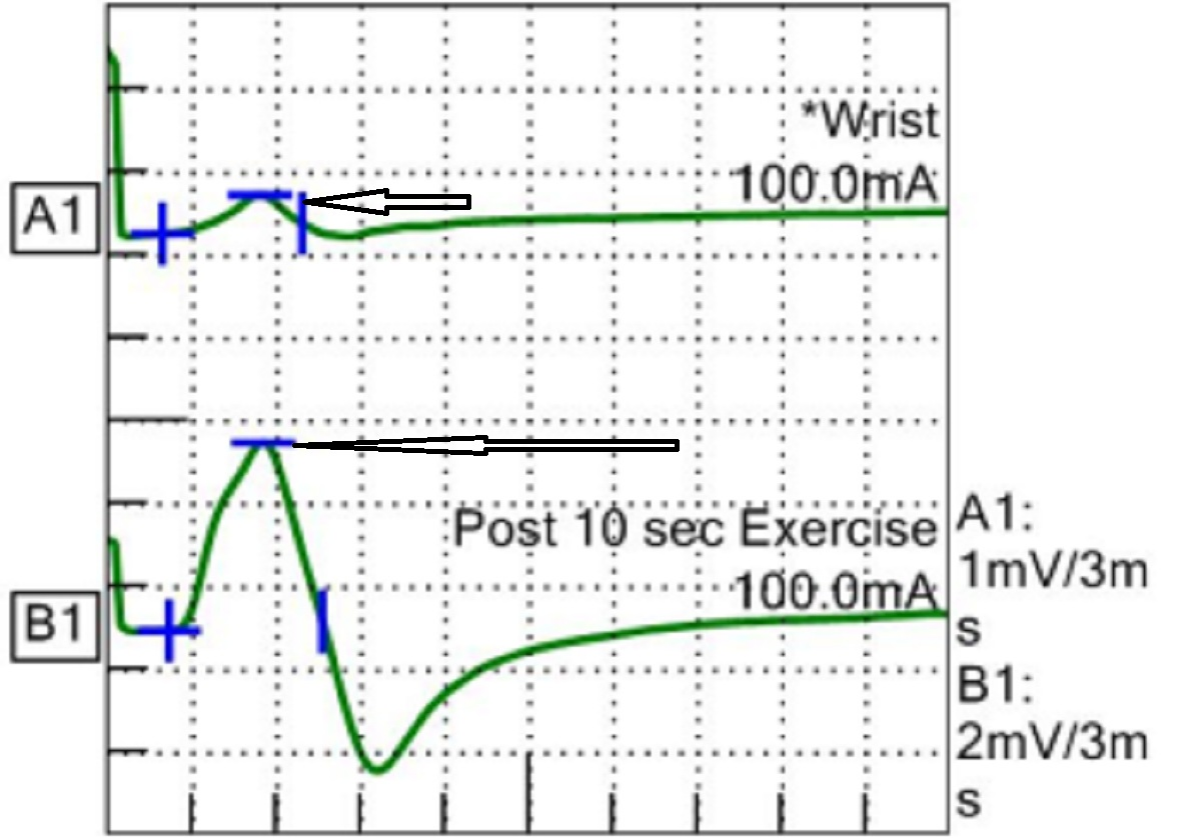
Improvement in compound motor action potential (CMAP).

On performing short exercise testing for 10 seconds her left ulnar CMAP increased to 10 mV (more than 100% increment) (Figure 4).

**Figure 5:**
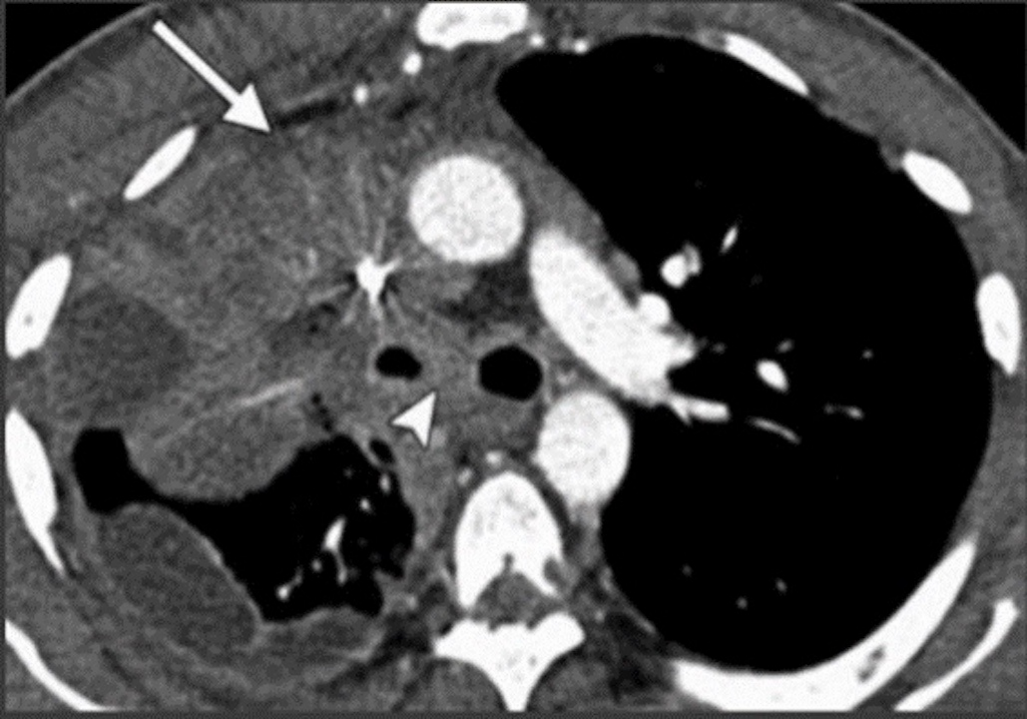
Computed tomography (CT) chest scan. CT chest shows a right upper lobe mass with mediastinal and carinal invasion, ipsilateral loculated pleural effusion, and thickening of the pleura.

This finding raised the suspicion of LEMS given her long-standing history of smoking. Computed tomography (CT) chest scan was ordered, which revealed precarinal/left lower para-tracheal mass with central necrosis (Figure 5).

On biopsy of the mass, small cell carcinoma was identified. On further evaluation, her P/Q antibody titer came out to be 3.62 nmol/L (normal <= 0.02, Mayo clinic), which confirmed the diagnosis of LEMS.

## Discussion

Our study highlights the importance of strong clinical suspicion in diagnosing patients with LEMS. The patient was initially diagnosed as having seronegative myasthenia in wake of her predominant ocular symptoms. Although rarely, ocular symptoms can be a presenting feature of LEMS and require high-degree of clinical suspicion for diagnosis. Important clues such as smoking, paraneoplastic feature (such as unexplained hyponatremia) should be extrapolated carefully from history. In our patient, the ocular symptoms were initially believed to be related to myasthenia, however long-standing history of smoking raised suspicion for possible underlying paraneoplastic phenomenon, which necessitated further evaluation with nerve conduction studies. On NCS of her left ulnar motor nerve, a reduced CMAP of 4.5 mV was noted along with 20% decrement in CMAP amplitude with slow repetitive nerve stimulation. Reduced CMAP amplitude although is a common finding seen in both myasthenia and Lambert–Eaton, but the latter was found as culprit. On performing short exercise testing for 10 seconds on her left ulnar nerve, CMAP increased to 10 mV (more than 100% increment) thus, correlating with hallmark NCS finding of Lambert–Eaton syndrome. Diagnosis of LEMS was confirmed with high P/Q antibody titer and finding of small cell carcinoma on CT chest. The underlying pathogenesis in LEMS is related to autoantibody formation against presynaptic voltage-gated calcium channel which disrupts neuromuscular transmission [2]. Majority of cases (60%) have underlying malignancy [3]. Weakness characteristically occurs in proximal limbs, although bulbar and ocular associations have also been reported [2]. Double vision, ptosis, and difficulty swallowing occur in such associations but are usually accompanied with proximal limb weakness in most of the presentations [2]. Previous studies have described the association of pure ocular weakness with diagnosis of LEMS. Rudnicki previously reported a case of a patient with LEMS and small cell lung carcinoma who presented with isolated ocular weakness [4]. Another study of 101 patients with LEMS reported associated ocular involvement. The frequency of ocular symptoms in this study increased from 5% at the onset of disease to 42% at six months. A total of 24% of the patients had diplopia, 24% had ptosis, 52% had both [4]. Although ocular symptoms are predominantly seen in myasthenia (59% of patients) [1], but such symptoms can also occur in LEMS, sometimes as the only presenting clinical symptom of the disease process. Diagnosis of LEMS in such cases can be challenging. Nonetheless, important clues in history indicating possible paraneoplastic involvement should be searched carefully in all patients with ocular presentation that can differentiate between two conditions and can aid greatly in providing correct diagnosis. A differential of LEMS should always be included in patients presenting with pure ocular weakness and suspicion should be confirmed with NCS. After an initial diagnosis of LEMS, efforts should be made to search the underlying malignancy. In majority of the cases (50-60%), CT chest scans can detect underlying malignancy instantaneously. Remaining malignancies are typically diagnosed within four years of LEMS diagnosis [2,3]. Treatment revolves around managing the malignancy, which can resolve symptoms of LEMS [2].

## Conclusions

Presence of unexplained hyponatremia or other paraneoplastic syndromes along with history of smoking should alert the possibility of LEMS even in those patients who have been diagnosed as ‘seronegative ocular myasthenia’. A differential of LEMS should be suspected in patients presenting with pure ocular weakness.
